# Impact of Comorbidities and Frailty on Early Shunt Failure in Geriatric Patients With Normal Pressure Hydrocephalus

**DOI:** 10.3389/fmed.2020.596270

**Published:** 2020-11-30

**Authors:** Alexis Hadjiathanasiou, Fatma Kilinc, Bedjan Behmanesh, Joshua Bernstock, Erdem Güresir, Muriel Heimann, Jürgen Konczalla, Elisa Scharnböck, Matthias Schneider, Leonie Weinhold, Volker Seifert, Hartmut Vatter, Florian Gessler, Patrick Schuss

**Affiliations:** ^1^Department of Neurosurgery, University Hospital Bonn, Bonn, Germany; ^2^Department of Neurosurgery, University Hospital Frankfurt, Frankfurt am Main, Germany; ^3^Department of Neurosurgery, Brigham and Women's Hospital, Boston, MA, United States; ^4^Institute for Medical Biometry, Informatics and Epidemiology (IMBIE), University Hospital Bonn, Bonn, Germany

**Keywords:** normal pressure hydrocephalus, post-operative complication, ventriculoperitoneal shunt, frailty, comorbidity, geriatric, shunt failure

## Abstract

**Background/Aim:** Older patients are considered to bear a higher perioperative risk. Since idiopathic normal pressure hydrocephalus (NPH) predominantly concerns older patients, identifying risk factors for early shunt failure for preoperative risk/benefit assessment is indispensable for indication and/or consultation of patients for ventriculoperitoneal shunting (VPS).

**Methods:** We performed a retrospective study design, including data acquired from two university hospital neurosurgical institutions between 2012 and 2019. Overall, 211 consecutive patients with clinical/radiological signs for NPH who additionally showed alleviation of symptoms after lumbar cerebrospinal fluid (CSF) drainage, received VPS and were included for further analysis. Frailty was measured using the Clinical Frailty Scale (CFS). Main outcome was early shunt failure or post-operative complications within 30 days after initial VPS surgery.

**Results:** The overall complication rate was 14%. Patient-related complications were observed in 13 patients (6%) and procedure-related complications in 16 patients (8%). Early post-operative complications resulted in a significantly prolonged length of hospital stay 6.9 ± 6.8 vs. 10.8 ± 11.8 days (*p* = 0.03). Diabetes mellitus with end-organ damage (OR 35.4, 95% CI 6.6 – 189.4, *p* < 0.0001) as well as preexisting Parkinson's disease were associated with early patient-related post-surgical complications after VPS for NPH.

**Conclusions:** Patients comorbidities but not frailty were associated with early post-operative patient-related complications in patients suffering NPH. While frailty may deter patients from other (neurosurgical) procedures, VPS surgery might contribute to treating NPH in these patients at a tolerable risk.

## Introduction

Normal pressure hydrocephalus (NPH) is a neurological syndrome in older patients characterized by ventriculomegaly and the clinical features of cognitive impairment, gait disturbances and/or urinary incontinence (1). Despite radiological signs of ventricular enlargement, patients suffering from NPH by definition do not exhibit increased intracranial pressure (ICP). The well-established treatment of NPH consists in the continuous drainage of cerebrospinal fluid (CSF), e.g., in the terms of a ventriculoperitoneal shunt (VPS). Given an adequate preoperative patient selection, an improvement of preoperative symptoms might be expected in up to 90% of cases (2). There are numerous, but not yet conclusive studies on the predominant fundamental discussions on potentially suitable valves and pressure settings to positively influence the neurological outcome and avoid long-term complications (2–5).

Despite its elective nature, VPS implantation is fraught with the risk of post-operative complications (infection, hygroma, under-, or overdrainage), which are reported to be as high as 38% (3, 4). The potentially positive effect of VP shunt implantation may be negated by the reported increased post-operative complication rate. To efficiently reduce the perioperative morbidity of VPS surgery in patients with NPH, a specific focus should also be placed on potential risk factors and thus avoid short-term post-operative complications. Further, the age distribution of NPH suggests that apparently older patients are most often affected (6). In addition to the increased likelihood of post-operative complications in older patients, complications in these patients may have a potentially higher impact due to a presumably reduced resilience (7). Next to age, scores measuring patient frailty are increasingly utilized to preoperatively identify patients potentially showing adverse events over the course of their treatment. Frailty is indicated by a decline in an individual's homeostatic reserves and results in an increased vulnerability to endogenous/exogenous stressors (8). Ultimately, despite several operational definitions of frailty, there is none that can serve as gold standard (9). Frailty attempts to quantify and measure biological age rather than relying on the purely chronological age (10). In various studies and as reviewed by Pazniokas et al. frailty has shown a superior predictive value of the decline in self-management when compared with the traditional metric of age (10, 11). Nevertheless, the literature on early post-operative complications/shunt failure in patients with NPH is scarce, in part due to different definitions of post-operative complications/adverse events.

In this study, we are examining the older patient cohort of two university hospitals to identify factors that may allow early detection of patients with NPH and an increased risk of post-operative complications/shunt failure.

## Methods

### Study Setting and Sample

A retrospective institutional database search was conducted to determine all VPS surgeries for NPH performed in older patients (aged ≥ 65 years) between January 2012 and December 2019 in two neurosurgical tertiary centers. Patients who underwent ventriculoatrial shunting, lumboperitoneal shunting, and/or endoscopic third ventriculostomy were excluded from further analysis.

Information, including patient characteristics on admission and during treatment course, radiological features, laboratory values, presenting symptoms, functional neurological status on admission, shunt valve, initial opening pressure setting, and occurrence of early post-operative complications, were collected and entered into a computerized database (SPSS, version 25, IBM Corp., Armonk, NY) after study approval by the local ethics committee. Patient comorbidities were assessed according to the Charlson comorbidity index (CCI) (12). To further evaluate the overall level of fitness/frailty in the geriatric patients studied, the Clinical Frailty Scale (CFS) was assessed as described previously (9). The CFS represents a judgment-based scale for assessing frailty. Its scores range from 1 (robust health) to 7 (complete functional dependence on others). Details of both the above assessments/frequencies are given in Supplementary Tables 1, 2.

### Diagnosis of NPH

As criteria for NPH the following criteria were required: a gradually developed gait disturbance with impairment of turning and stride length/width, urinary dysfunction, a mild to moderate cognitive impairment with memory deficits and/or reduced alertness, a systematic ventriculomegaly without clinically relevant parenchymal lesions, a lack of occlusion in the ventricular system and an Evans' index > 0.30. Subsequently, patients underwent lumbar CSF drainage (5–10 ml/h) for 3 days or spinal tap test (STT). Thereafter, the above-mentioned criteria were clinically re-evaluated. In the case of an objectively detectable improvement (gait pattern, psychometric functions), implantation of VPS was indicated. For further analysis, patients with NPH were divided into two groups including patients with typical complete Hakim triad symptoms (gait disturbance, cognitive impairment, and urinary dysfunction) and patients with incomplete Hakim triad symptoms.

### VPS Surgery

All patients with NPH who underwent ventriculoperitoneal shunt implantation were included in further analysis. VPS was placed primarily in the right frontal horn as previously reported (13–15). The accuracy of ventricular catheter placement was evaluated post-operatively as previously described (16). Programmable (Hakim-Medos/Certas, Johnson & Johnson) as well as non-programmable shunt valves (OSV II, Integra Neurosciences) were implanted in patients with NPH. Furthermore, an additional antisiphon device (ASD) was implanted alongside with the VPS in some patients. The decision on the shunt valve/ASD depended on the preference of the treating neurosurgeon and the discretion/standard operating procedure of the collaborating centers. The threshold values for the opening pressure of gravitational units/programmable valves were also decided at the discretion of the respective collaborating centers. The manufacturers' recommendations were followed throughout the treatment process. Each patient received a post-operative cranial CT scan to verify the correct position of the intracranial catheter as well as X-ray scans of head/neck, chest and abdomen to ensure correct shunt alignment and intraabdominal catheter positioning.

### Definition of Post-operative Complication

Outcome of interest in the present study were short-term post-operative complications or shunt failure defined as any adverse event that resulted in a surgical procedure to revise the shunt and/or address the complication. Only complications/adverse events observed within the first 30 days after VPS surgery were included in the present analysis. Early post-operative complications were further categorized as procedure-related (malposition, material failure) and patient-related complications (bleeding, infection). For further exploratory analysis regarding potential influencing factors, only patient-related complications were considered. Procedure-related complications are presented hereafter solely for the purpose of full coverage.

### Statistics

Data analyses were performed using the computer software package SPSS (version 25, IBM Corp., Armonk, NY). As the continuous variables could not be assumed to be normally distributed (graphical inspection), Mann–Whitney *U*-tests were used to compare patients without early post-operative complications with patients with early post-operative complications. Categorical variables were analyzed in contingency tables using Fisher's exact test. Results with *p* < 0.05 were considered statistically significant. Patients with incomplete data sets were excluded from further analysis. An additional exploratory multivariate analysis, a binary logistic regression was conducted to determine independent predictors of patient-related post-operative complications in older patients with NPH. The covariate selection was determined by available knowledge (17) and included the following potential confounders: sex (18), anticoagulant medication prior surgery (19), diabetes mellitus (20), pre-existing Parkinson's disease (21), and surgery beginning before 10 a.m. (22). To specifically investigate of the association between frailty and patient-related post-operative complications, we additionally performed an ordinal regression analysis.

## Results

### Patient Characteristics

Between 2012 and 2019, 211 geriatric patients suffering from NPH underwent VPS surgery in both facilities. Of those, 114 (54%) patients with NPH and subsequent VPS surgery presented with complete Hakim triad symptoms, while 97 (46%) patients presented with an incomplete symptom triad complex. The mean Evans' index was 0.37 ± 0.07. Further details on patients with NPH and VPS surgery are given in Table 1.

**Table 1:** Baseline patient characteristics.

**No. of patients**	**211**
Female sex (%)	84 (40%)
Mean age (yrs)	76 ± 7
Complete hakim triad symptoms	114 (54%)
Mean evans index	0.37 ± 0.07
ASA score 1 or 2	139 (66%)
**Charlson Comorbidity Index (%)**	
0	91 (43%)
1	45 (21%)
2	39 (19%)
3	15 (7%)
4	12 (6%)
>4	9 (4%)
Clinical frailty scale >5	40 (19%)
**Shunt valve**	
ProGAV	68 (32%)
Certas	50 (24%)
Others	29 (14%)
Fixed shunt valves	64 (30%)
Antisiphon device	68 (32%)
Mean duration of surgery (min)	56 ± 19
Median length of stay (days)	6 ± 7.8
Early post-operative complication (%)	29 (14%)

### Patient Frailty

**Figure 1:**
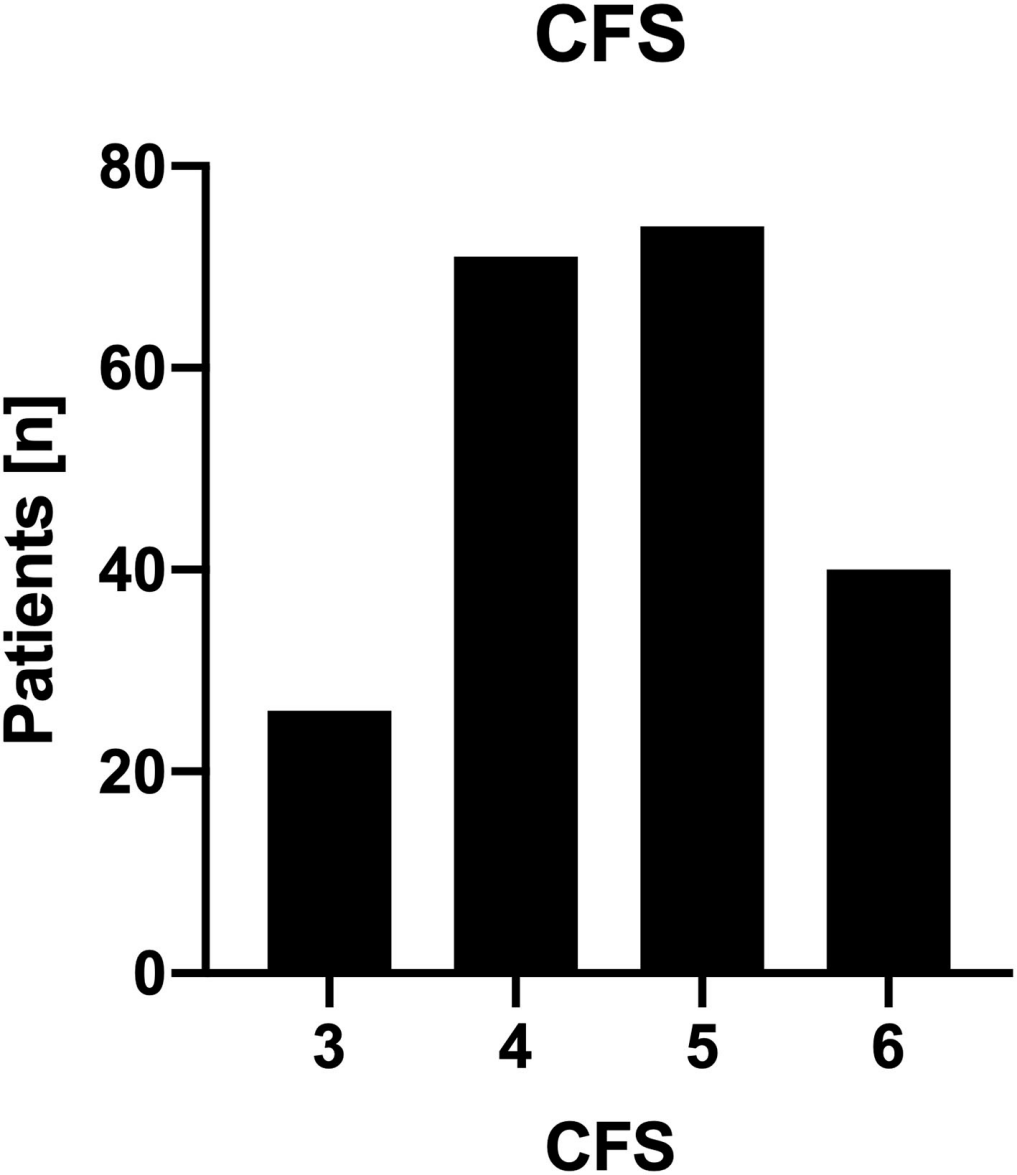
Distribution of patient CFS. Histogram of clinical frailty score. This histogram demonstrates the distribution of patient CFS among patients undergoing surgery for VPS placement to treat NPH.

Patient frailty was assessed as described in the methods section of the manuscript. All patients (100%) displayed at least some degree of frailty (CFS ≥ 3). Of note, 40 patients (19%) displayed a significant degree of frailty with a CFS of 6 indicating help with all outside activities and with keeping house. Detailed information on the degree of frailty is given in Figure 1.

### Early Post-operative Complications

Overall, 29 out of 211 patients (14%) with NPH developed an early post-operative complication leading to shunt failure within the first 30 days after initial VPS surgery. In detail, post-operative complication with shunt failure consisted of VPS infection/meningitis in 5 patients (2%), intraparenchymal hemorrhage in 4 patients (2%), subdural hematoma/hygroma in 3 patients (1%), and injury to the intestines in 1 patient (<1%). Further details are presented in Table 2. Early post-operative complications after VPS surgery for NPH occurred after a median of 12 days after the initial surgery and resulted in a significantly longer length of hospital stay (6.9±6.8 days vs. 10.8±11.8 days; *p* = 0.03).

**Table 2:** Early post-operative complications with surgical consequences.

**Overall complications (%)**	**29 (14)**
**Patient-related complications (%)**	**13 (6)**
VPS infection/meningitis	5
Intraparenchymal hemorrhage	4
Subdural hematoma/hygroma	3
Visceral injury	1
**Procedure-related complications (%)**	**16 (8)**
Misplacement/migration abdominal catheter	11
Misplacement intracranial catheter	3
Mechanical dysfunction	2

*The bold values are intended to differentiate between two kind of complications. The numbers referee to cases and in brackets is shown the percentage*.

### Influence of Perioperative Parameters

Routine preoperative laboratory tests indicated no association with early post-operative complications (data not shown).

The choice of the implanted valve did not influence the early post-operative complication rate. In detail, no significant difference in early post-operative complication was observed between patients undergoing implantation of a fixed or an adjustable valve were observed. Further, no significant association between the different adjustable valves with early post-operative complications was observed (*p* = 0.8). The duration of VPS surgery did not differ between patients with or without early post-operative complications (*p* = 0.7). No difference was observed between patients undergoing VPS surgery at the initial case of a day or later during the day regarding early complications (*p* = 0.4).

The catheter position was assessed using the Hayhurst shunt grading system at the post-operative computed tomography (CT). Overall, 155 (73%) ventricular catheters were positioned optimally with free-floating in CSF, 50 (24%) touching the choroid or ventricular wall and 6 (3%) intraparenchymal. Revision surgery was carried out in three cases with intraparenchymal position (Hayhurst grade 3). In the other three cases, no revision surgery was carried out, because of a partial contact of the catheter to the CSF and sustained functionality.

### Influence of Comorbidity Burden

Despite the focus on VPS surgery, a previous abdominal surgery did not have a significant impact on the post-operative complication rate.

Neither a higher CCI nor an age adjusted CCI was associated with a higher rate of post-operative complications (Table 3). American society of anesthesiologists physical score (ASA) indicated no influence on post-operative complications. Most patients (66%) presented with an ASA score of I-II and 72 (34%) patients with an ASA score of III. None of the patients treated presented with an ASA score IV.

**Table 3:** Comparison of patients with and w/o early post-operative complications.

	**Patients w/o early post-operative complications**	**Patients with early post-operative complications**	***p*-value**
**No. of patients (%)**	**182 (86)**	**29 (14)**	
Female sex (%)	70 (39)	14 (48)	*p* = 0.32
Mean age (yrs)	75 ± 7	76 ± 6	*p* = 0.37
Complete hakim triad symptoms (%)	101 (56)	13 (45)	*p* = 0.32
Mean Evans Index	0.37 ± 0.07	0.36 ± 0.08	*p* = 0.22
ASA score 1 or 2 (%)	123 (68)	16 (55)	*p* = 0.21
Age-adjusted charlson comorbidity index >4 (%)	64 (35)	15 (52)	*p* = 0.21
Clinical frailty scale >5 (%)	32 (18)	8 (28)	*p* = 0.21
Pre-existing Parkinson's disease (%)	8 (4)	2 (7)	*p* = 0.63
Diabetes mellitus with end-organ damage (included in CCI, %)	1 (0.5)	6 (21)	OR 47.2, 95%CI 5.4-409.8, *p* < 0.0001
Previous abdominal surgery (%)	34 (19)	6 (21)	*p* = 0.8
**Shunt valve**			*p* = 0.83
Adjustable (%)	126 (69)	21 (72)	
Fixed shunt valves (%)	56 (31)	8 (18)	
ASD (%)	58 (32)	10 (35)	*p* = 0.83
Surgery beginning before 10 a.m. (%)	110 (60)	20 (69)	*p* = 0.25
Mean duration of surgery (min)	57.1 ± 19.6	58.7 ± 16.1	*p* = 0.38
Hayhurst grade 1 (%)	133 (73)	22 (76)	*p* = 0.82
Median length of stay (days)	6.9 ± 6.8	10.8 ± 11.8	*p* = 0.03

In total, 70 NPH patients (33%) suffered from pre-existing diabetes mellitus (DM) of which 7 patients presented with DM associated with end-organ damage (3%). Patients with early post-operative complications suffered significantly more often from DM with end-organ damage when compared to patients without post-operative complications (21 vs. 0.5%, OR 47.2, 95% CI 5.4–410.1, *p* < 0.001, Table 3).

### Multivariate Analysis

We performed a multivariate logistic regression analysis of those variables that were considered to be potentially associated with patient-related complications based on available knowledge. The multivariate analysis revealed “diabetes mellitus with end-organ damage” (OR 35.4; 95% CI 6.6–189.4; *p* < 0.0001) as well as “pre-existing Parkinson's disease” (OR 6.6; 95% CI 1.2–37.2; *p* = 0.03) as significant and independent predictors for early post-operative patient-related complications after VPS surgery for NPH (Nagelkerke's *R*^2^ 0.22). Ordinal regression analysis revealed no significant association of patient-related post-operative complication with severity of frailty according to the CFS (OR = 0.982, CI 0.355–2.721, *p* = 0.973).

## Discussion

VPS placement is the most established surgical treatment of NPH. Although published improvement rates after such surgery are high, some studies cautioned against a potentially significant complication rate (2–4). Therefore, great interest arises to identify possible factors that predict early VPS failure and post-operative complications. These have a great impact on this particular patient population, as it is an elderly, sometimes highly pre-morbid group of patients. Due to the expected increased probability of complications, a better risk/benefit assessment of VPS surgery in older NPH patients is therefore possible if potential risk factors are known.

Complications after shunt surgery can be differentiated between symptomatic and only radiologically diagnosed, necessity of surgical (re-)intervention, early (occurring in the first 30 d) and late. The EU-iNPH study (n = 142) revealed that a large proportion of complications appear to occur within the first post-operative month (23). The same study reported an overall complication rate of 28%, with hygromas and SDH occurring in 9 and 6%, respectively. A systematic review of the literature with 30 included studies (performed after 2006) and 1,573 patients reported an overall complication rate of 8.2% (24). We present comparable results when non-patient-related misplacement complications are excluded (6%).

To provide a solid conclusion that excludes potentially confusing factors that could result from a long observation period in NPH patients, the present analysis is limited to this first month defining complication as the necessity of a surgical treatment.

### Procedure-Related Complications

One common problem in shunt patients is symptomatic under- or overdrainage. Antisiphon/antigravity devices (ASD) and adjustable valves (32% in the present study) played an important role in reducing the amount of problems associated with overdrainage. However, in the present study there was no statistical difference in the development of SDH as a sign of overdrainage in patients with or without ASD. This finding is in contrast to the results from the SVASONA study (Shunt Valves Plus Shunt Assistant vs. Shunt Valves Alone for Controlling Overdrainage in Idiopathic Normal Pressure Hydrocephalus in Adults) (5). Nevertheless, Gasslander et al. recently reported no statistical difference (*p* > 0.99) comparing SDH rates in NPH patients with and without ASD in 1,457 patients, which is consistent with the results of the present study (25). In addition, in some patients with NPH, the presence of a thin, non-symptomatic hygroma may be acceptable to maximize the benefit of VPS implantation (23).

The high rate of misplacement of the implanted material in this two center-study represents avoidable complications that cannot be attributed to possible risk factors of the individual patient. Nevertheless, this observation may also be a consequence of the systematic post-operative imaging controls carried out in all NPH patients, as asymptomatic patients are also detected and possibly revised in this way.

### Patient-Related Complications: Diabetes Mellitus

Diabetes mellitus (DM) is a known cause of complications in the microvascular system leading to an increased risk of acute post-operative complications (19). Diabetic patients are at higher risk of infection due to impaired immune function secondary to hyperglycemia. Studies have demonstrated decreased granulocyte function and microbicidal action in diabetics (26). A recent literature review speculates on a possible ventriculomegaly-induced pituitary dysfunction, which may allow the later development of DM and thus could explain the described increased incidence of DM in the NPH population (27). In a finish cohort study, DM was found to be overrepresented in patients with NPH and more often associated with mortality (28). In the current study, DM was detected preoperatively in one third of geriatric patients (33%). Furthermore, when DM caused end-organ damage, early post-surgical patient-related complications occurred significantly more often according to the performed multivariate analysis (OR 47.2, 95% CI 5.4–410.1; *p* < 0.001).

### Patient-Related Complications: Frailty

To our knowledge, this is the first study specifically addressing the impact of frailty on the post-operative course of patients suffering from NPH undergoing VPS surgery (10). The present analysis demonstrated no significant association between frailty and patient-related complications after VPS surgery for NPH in older patients. Within the literature on NPH, the potential influence of frailty has been almost neglected. Nevertheless, a recent study in a cohort of older adults with NPH identified a significant association between central nervous system biomechanical response and frailty. Frailty therefore does not appear to be simply a static feature, but part of a dynamic process (29). Others have studied the predictive value of frailty in comparison with other variables such as age, ASA score or CCI: while frailty may indicate an increased risk for the development of complications after surgery for neurosurgical disease (10). Ondeck et al. described that ASA and age were equivalent or better in predicting post-operative adverse outcomes (30). Further, Shimizu et al. found that age was a better predictor of poor outcome after subdural bleeding than frailty (31). As we did not operate on patients without any signs of frailty or patients with excessive frailty (i.e., CFS ≥ 7), these results may reflect the highly selected patients undergoing VPS surgery for NPH in our institutions. The true effects of frailty on the incidence of post-operative complications, especially in patients with an excessive degree of frailty need to be separately addressed, if possible, in a prospective trial. However, aside from NPH the many different measurement scales and definitions of frailty pose an additional obstacle as they impede the comparability of clinical studies (8).

## Limitations

The present study has several limitations. Acquisition of data was carried out retrospectively. Patients were not randomized but treated according to the preference of the treating neurosurgeon leading to a high risk for selection bias. The use of CFS provides an individually adaptable tool for the assessment of frailty, since it belongs to the judgement-based scales regarding frailty. For this subjective judgment a certain degree of professional routine in dealing with older people is mandatory. Since the assessment in this study was not performed by geriatric specialists, the subjectivity of CFS must be mentioned as a potential limitation and source of error. In this study, only clinically relevant early post-operative complications requiring reintervention within 30 days were examined. Late complications occurring during the later course of the disease or treatment failure were therefore not included. Furthermore, the limited number of patient-related complications interferes with covariate selection for multivariate analysis. However, it is precisely this strict definition of the post-operative complication and the focus on patient-related complications that provides for comparability without other significant confounding factors. Nevertheless, the results of this study and further post-operative periods of time will need to be clarified in further studies.

## Conclusions

This study provides insight into potential risk factors that could alter resource utilization and post-operative care to prevent early post-operative complications in selected NPH patients. Certain comorbidities but not frailty in patients with NPH were associated with early post-operative patient-related complications. While frailty may deter patients from other (neurosurgical) procedures, VPS surgery might contribute to treating NPH in these patients at a tolerable risk. In providing care to these patients and not refraining from surgery, neurosurgeons may improve the lives of both patients and their families.

## Data Availability Statement

The raw data supporting the conclusions of this article will be made available by the authors, without undue reservation.

## Ethics Statement

The studies involving human participants were reviewed and approved by Ethics committee of the University Hospital Bonn. Written informed consent for participation was not required for this study in accordance with the national legislation and the institutional requirements.

## Author Contributions

AH: study design, data acquisition, wrote, and reviewed the manuscript. FK: data acquisition, wrote, and reviewed the manuscript. BB: reviewed the manuscript. EG: reviewed the manuscript. MH: data acquisition. JK: reviewed the manuscript. ES: data acquisition. MS: data acquisition and reviewed the manuscript. LW: statistical analysis. VS: reviewed the manuscript. HV: reviewed the manuscript. FG: data acquisition, wrote and reviewed the manuscript, and study supervision. PS: conceptualized the manuscript, data design, statistical analysis, wrote and reviewed the manuscript, and study supervision. All authors contributed to the article and approved the submitted version.

## Conflict of Interest

The authors declare that the research was conducted in the absence of any commercial or financial relationships that could be construed as a potential conflict of interest.

## References

[1] Hakim S Adams RD. The special clinical problem of symptomatic hydrocephalus with normal cerebrospinal fluid pressure. Observations on cerebrospinal fluid hydrodynamics. J Neurol Sci. (1965) 2:307–27. 10.1016/0022-510X(65)90016-X 5889177

[2] Poca MA Solana E Martinez-Ricarte FR Romero M Gandara D Sahuquillo J. Idiopathic normal pressure hydrocephalus: results of a prospective cohort of 236 shunted patients. Acta Neurochir Suppl. (2012) 114:247–53. 10.1007/978-3-7091-0956-4_49 22327703

[3] Hebb AO Cusimano MD. Idiopathic normal pressure hydrocephalus: a systematic review of diagnosis and outcome. Neurosurgery. (2001) 49:1166–84. 10.1227/00006123-200111000-00028 11846911

[4] Nadel JL Wilkinson DA Linzey JR Maher CO Kotagal V Heth JA. Thirty-day hospital readmission and surgical complication rates for shunting in normal pressure hydrocephalus: a large national database analysis. Neurosurgery. (2020) 86:843–50. 10.1093/neuros/nyz299 31420654 PMC7528659

[5] Lemcke J Meier U Muller C Fritsch MJ Kehler U Langer N. Safety and efficacy of gravitational shunt valves in patients with idiopathic normal pressure hydrocephalus: a pragmatic, randomised, open label, multicentre trial (SVASONA). J Neurol Neurosurg Psychiatry. (2013) 84:850–7. 10.1136/jnnp-2012-303936 23457222 PMC3717598

[6] Jaraj D Rabiei K Marlow T Jensen C Skoog I Wikkelso C. Prevalence of idiopathic normal-pressure hydrocephalus. Neurology. (2014) 82:1449–54. 10.1212/WNL.0000000000000342 24682964 PMC4001197

[7] Sieber FE Barnett SR. Preventing postoperative complications in the elderly. Anesthesiol Clin. (2011) 29:83–97. 10.1016/j.anclin.2010.11.011 21295754 PMC3073675

[8] Cesari M Calvani R Marzetti E. Frailty in older persons. Clin Geriatr Med. (2017) 33:293–303. 10.1016/j.cger.2017.02.002 28689563

[9] Rockwood K Song X MacKnight C Bergman H Hogan DB McDowell I. A global clinical measure of fitness and frailty in elderly people. CMAJ. (2005) 173:489–95. 10.1503/cmaj.050051 16129869 PMC1188185

[10] Pazniokas J Gandhi C Theriault B Schmidt M Cole C Al-Mufti F. The immense heterogeneity of frailty in neurosurgery: a systematic literature review. Neurosurg Rev. (2020). 10.1007/s10143-020-01241-2. [Epub ahead of print]. 31953785

[11] Kim SW Han HS Jung HW Kim KI Hwang DW Kang SB. Multidimensional frailty score for the prediction of postoperative mortality risk. JAMA Surg. (2014) 149:633–40. 10.1001/jamasurg.2014.241 24804971

[12] Charlson ME Pompei P Ales KL MacKenzie CR. A new method of classifying prognostic comorbidity in longitudinal studies: development and validation. J Chronic Dis. (1987) 40:373–83. 10.1016/0021-9681(87)90171-8 3558716

[13] Ilic I Schuss P Borger V Hadjiathanasiou A Vatter H Fimmers R. Ventriculostomy with subsequent ventriculoperitoneal shunt placement after subarachnoid hemorrhage: the effect of implantation site on postoperative complications-a single-center series. Acta Neurochir. (2020) 162:1831–6. 10.1007/s00701-020-04362-1 32415487

[14] Schuss P Borger V Güresir Á Vatter H Güresir E. Cranioplasty and ventriculoperitoneal shunt placement after decompressive craniectomy: staged surgery is associated with fewer postoperative complications. World Neurosurg. (2015) 84:1051–4. 10.1016/j.wneu.2015.05.066 26072458

[15] Won SY Dubinski D Behmanesh B Bernstock JD Seifert V Konczalla J. Management of hydrocephalus after resection of posterior fossa lesions in pediatric and adult patients-predictors for development of hydrocephalus. Neurosurg Rev. (2019) 43:1143–50. 10.1007/s10143-019-01139-8 31286305

[16] Hayhurst C Beems T Jenkinson MD Byrne P Clark S Kandasamy J. Effect of electromagnetic-navigated shunt placement on failure rates: a prospective multicenter study. J Neurosurg. (2010) 113:1273–8. 10.3171/2010.3.JNS091237 20397892

[17] Lederer DJ Bell SC Branson RD Chalmers JD Marshall R Maslove DM. Control of confounding and reporting of results in causal inference studies. Guidance for authors from editors of respiratory, sleep, and critical care journals. Ann Am Thorac Soc. (2019) 16:22–8. 10.1513/AnnalsATS.201808-564PS 30230362

[18] Meier U Stengel D Muller C Fritsch MJ Kehler U Langer N. Predictors of subsequent overdrainage and clinical outcomes after ventriculoperitoneal shunting for idiopathic normal pressure hydrocephalus. Neurosurgery. (2013) 73:1054–60. 10.1227/NEU.0000000000000155 24257332

[19] Schuss P Güresir Á Schneider M Velten M Vatter H Güresir E. Factors influencing early postoperative complications following surgery for symptomatic spinal metastasis: a single-center series and multivariate analysis. Neurosurg Rev. (2020) 43:211–6. 10.1007/s10143-018-1032-3 30219955

[20] Yang YN Zhang J Gu Z Song YL. The risk of intracranial infection in adults with hydrocephalus after ventriculoperitoneal shunt surgery: a retrospective study. Int Wound J. (2020) 17:722–8. 10.1111/iwj.13331 32073232 PMC7949404

[21] Kiefer M Eymann R Steudel WI. Outcome predictors for normal-pressure hydrocephalus. Acta Neurochir Suppl. (2006) 96:364–7. 10.1007/3-211-30714-1_75 16671486

[22] Korinek AM Fulla-Oller L Boch AL Golmard JL Hadiji B Puybasset L. Morbidity of ventricular cerebrospinal fluid shunt surgery in adults: an 8-year study. Neurosurgery. (2011) 68:985–94. 10.1227/NEU.0b013e318208f360 21221037

[23] Feletti A d'Avella D Wikkelso C Klinge P Hellstrom P Tans J. Ventriculoperitoneal shunt complications in the European idiopathic normal pressure hydrocephalus multicenter study. Oper Neurosurg. (2019) 17:97–102. 10.1093/ons/opy232 30169650

[24] Toma AK Papadopoulos MC Stapleton S Kitchen ND Watkins LD. Systematic review of the outcome of shunt surgery in idiopathic normal-pressure hydrocephalus. Acta Neurochir. (2013) 155:1977–80. 10.1007/s00701-013-1835-5 23975646

[25] Gasslander J Sundstrom N Eklund A Koskinen LD Malm J. Risk factors for developing subdural hematoma: a registry-based study in 1457 patients with shunted idiopathic normal pressure hydrocephalus. J Neurosurg. (2020). 10.3171/2019.10.JNS191223. [Epub ahead of print]. 31923893

[26] Bagdade JD Stewart M Walters E. Impaired granulocyte adherence. A reversible defect in host defense in patients with poorly controlled diabetes. Diabetes. (1978) 27:677–81. 10.2337/diab.27.6.677 658613

[27] Hudson M Nowak C Garling RJ Harris C. Comorbidity of diabetes mellitus in idiopathic normal pressure hydrocephalus: a systematic literature review. Fluids Barriers CNS. (2019) 16:5. 10.1186/s12987-019-0125-x 30744635 PMC6371499

[28] Pyykko OT Nerg O Niskasaari HM Niskasaari T Koivisto AM Hiltunen M. Incidence, comorbidities, and mortality in idiopathic normal pressure hydrocephalus. World Neurosurg. (2018) 112:e624–e31. 10.1016/j.wneu.2018.01.107 29374607

[29] Vallet A Del Campo N Hoogendijk EO Lokossou A Baledent O Czosnyka Z. Biomechanical response of the CNS is associated with frailty in NPH-suspected patients. J Neurol. (2020) 267:1389–400. 10.1007/s00415-019-09689-z 31997040

[30] Ondeck NT Bohl DD Bovonratwet P McLynn RP Cui JJ Shultz BN. Discriminative ability of commonly used indices to predict adverse outcomes after poster lumbar fusion: a comparison of demographics, ASA, the modified Charlson comorbidity index, and the modified frailty index. Spine J. (2018) 18:44–52. 10.1016/j.spinee.2017.05.028 28578164

[31] Shimizu K Sadatomo T Hara T Onishi S Yuki K Kurisu K. Importance of frailty evaluation in the prediction of the prognosis of patients with chronic subdural hematoma. Geriatr Gerontol Int. (2018) 18:1173–6. 10.1111/ggi.13436 29770549

