# Supplementary material for: Impact of Comorbidities and Frailty on Early Shunt Failure in Geriatric Patients With Normal Pressure Hydrocephalus
Source: Front Med (Lausanne). 2020 Nov 30;7:596270. doi: 10.3389/fmed.2020.596270 (PMC7734184; doi:10.3389/fmed.2020.596270)
Supplement: Supplementary file 1 [file Data_Sheet_1.docx]

**Supplementary eTable 1** - Frequency of Charlson Comorbidity Index Conditions (n=211).

**Supplementary eTable 2** - Frequency of patient frailty according to the Clinical Frailty Scale (n=211).

**Supplementary eTable 1** - Frequency of Charlson Comorbidity Index Conditions (n=211).

| **Index weight** | **Condition** | **Frequency % (n)** |
| --- | --- | --- |
| 1 | coronary artery disease | 9.5 (20) |
| 1 | congestive heart failure | 8.1 (17) |
| 1 | peripheral vascular disease | 1.9 (4) |
| 1 | cerebrovascular disease | 10 (21) |
| 1 | dementia | 69.2 (146) |
| 1 | chronic pulmonary disease | 4.3 (9) |
| 1 | connective tissue disease | 0.9 (2) |
| 1 | ulcer disease | 3.8 (8) |
| 1 | mild liver disease | 0.9 (2) |
| 1 | diabetes | 29.9 (63) |
| 2 | hemiplegia | 0.9 (2) |
| 2 | moderate / severe renal disease | 5.7 (12) |
| 2 | diabetes with end-organ damage | 3.3 (7) |
| 2 | any tumor | 11.4 (24) |
| 2 | leukemia | 0.9 (2) |
| 2 | lymphoma | 1.4 (3) |
| 3 | moderate / severe liver disease | 0.5 (1) |
| 6 | metastatic solid tumor | 0 (0) |
| 6 | AIDS | 0 (0) |

**Supplementary eTable 2** - Frequency of patient frailty according to the Clinical Frailty Scale (n=211).

| **Category** | **Description** | **Frequency % (n)** |
| --- | --- | --- |
| 1 | very fit - robust, active, energetic, well motivated and fit; these people usually exercise regularly and belong to the group best suited to their age | 0 (0) |
| 2 | well - without active disease, but are less fit than people in category 1 | 0 (0) |
| 3 | well, with treated comorbid disease - disease symptoms are adequately controlled compared to those of category 4 | 12.3 (26) |
| 4 | apparently vulnerable - although they are not explicitly dependent, these people often express complaints that they are "slowed down" and/or have symptoms of illness | 33.6 (71) |
| 5 | mildy frail - having limited dependence on others for instrumental activities of daily living (IADL) | 35.1 (74) |
| 6 | moderately frail - help is needed for both IADLs and non-IADLs | 19 (40) |
| 7 | severely frail - completely dependent on others for all activities of daily life and/or terminally ill | 0 (0) |
